# Non-conventional and Investigational PET Radiotracers for Breast Cancer: A Systematic Review

**DOI:** 10.3389/fmed.2022.881551

**Published:** 2022-04-12

**Authors:** Michele Balma, Virginia Liberini, Manuela Racca, Riccardo Laudicella, Matteo Bauckneht, Ambra Buschiazzo, Daniele Giovanni Nicolotti, Simona Peano, Andrea Bianchi, Giovanni Albano, Natale Quartuccio, Ronan Abgral, Silvia Daniela Morbelli, Calogero D'Alessandria, Enzo Terreno, Martin William Huellner, Alberto Papaleo, Désirée Deandreis

**Affiliations:** ^1^Nuclear Medicine Department, S. Croce e Carle Hospital, Cuneo, Italy; ^2^Division of Nuclear Medicine, Department of Medical Science, University of Turin, Turin, Italy; ^3^Nuclear Medicine Unit, Candiolo Cancer Institute, FPO-IRCCS, Candiolo, Italy; ^4^Department of Biomedical and Dental Sciences and of Morpho-Functional Imaging, Nuclear Medicine Unit, University of Messina, Messina, Italy; ^5^Department of Nuclear Medicine, University Hospital Zurich, University of Zurich, Zurich, Switzerland; ^6^Nuclear Medicine Unit, Fondazione Istituto G. Giglio, Cefalù, Italy; ^7^IRCCS Ospedale Policlinico San Martino, Genoa, Italy; ^8^Department of Health Science (DISSAL), University of Genoa, Genoa, Italy; ^9^Nuclear Medicine Unit, A.R.N.A.S. Civico di Cristina and Benfratelli Hospitals, Palermo, Italy; ^10^Department of Nuclear Medicine, University Hospital of Brest, Brest, France; ^11^Department of Nuclear Medicine, Klinikum Rechts der Isar TU München, Munich, Germany; ^12^Department of Molecular Biotechnology and Health Sciences, Molecular & Preclinical Imaging Centers, University of Turin, Turin, Italy

**Keywords:** PET, breast cancer, radiotracers, molecular imaging, FES, FLT, FAPI, PSMA

## Abstract

Breast cancer is one of the most common malignancies in women, with high morbidity and mortality rates. In breast cancer, the use of novel radiopharmaceuticals in nuclear medicine can improve the accuracy of diagnosis and staging, refine surveillance strategies and accuracy in choosing personalized treatment approaches, including radioligand therapy. Nuclear medicine thus shows great promise for improving the quality of life of breast cancer patients by allowing non-invasive assessment of the diverse and complex biological processes underlying the development of breast cancer and its evolution under therapy. This review aims to describe molecular probes currently in clinical use as well as those under investigation holding great promise for personalized medicine and precision oncology in breast cancer.

## Introduction

Breast cancer (BC) is a very heterogeneous disease. It is the most common malignancy in women, accounting for ~30% of female cancers worldwide. Female BC has now an estimated 2.3 million new cases per year, representing 11.7% of all cancer cases in 2020. It is the fifth leading cause of cancer mortality worldwide, with 685,000 deaths per year. The worldwide incidence varies between 29.7 per 100,000 (transitioning countries) and 55.9 per 100,000 (transitioned countries), reflecting the association between BC incidence and the degree of economic development and associated social and lifestyle factors (1).

The disease is considered curable when it is confined to the breast or in case of spread only to axillary lymph nodes, defined as early breast cancer. Here, treatment is curative in ~70–80% of cases, also owing to the development of new therapeutic strategies. Conversely, advanced disease is not considered curable, but treatable, and primary goals of therapy are prolonging survival, controlling symptoms, and improving quality of life (2).

Therefore, an accurate staging of BC is critical for its clinical and therapeutic management. In clinical practice, ultrasonography, mammography, and magnetic resonance imaging (MRI) are mainly used to assess the local disease extent. In order to assess the presence of distant metastases, whole-body imaging is required. Although radiological imaging, such as computed tomography (CT) and MRI, is still more widely used for this purpose, in recent years nuclear medicine imaging has also gained more importance, thanks to the use of positron emission tomography (PET), PET/CT or PET/MRI. In particular, 2-deoxy-2-[^18^F]fluoro-D-glucose ([^18^F]F-FDG) PET is helpful in identifying otherwise undetected distant metastases in advanced BC (3–5). However, several factors can result in false-negative [^18^F]F-FDG imaging in BC, owing, e.g., to the small size of a tumor or to its molecular and histological characteristics (6).

## Breast Cancer Biology and Classification

Regarding BC biology and classification, invasive breast cancer (IBC) encompasses a broad spectrum of histological subtypes, with the World Health Organization (WHO) recognizing at least 18 histologically different varieties. Invasive breast cancer of no special type (IBC-NST), previously known as invasive ductal carcinoma, is the most frequent subgroup, accounting for 40–80% of cases. The second most common histological subtype is invasive lobular breast carcinoma (ILBC) (7).

In recent years, the advancement and widespread application of “omics” technologies (genomics, epigenomics, transcriptomics, or proteomics, among others) has led to new discoveries based mainly on morphological and immunohistochemical characterization of the tumor, but also on genetic profiling of the tumor. In 10% of BC cases, there is a genetic predisposition and the most commonly associated germline mutations are *breast cancer gene 1* (BRCA1) and *breast cancer gene 2* (BRCA2) (8).

The most important biomarkers of BC are estrogen receptor (ER), progesterone receptor (PR), Ki-67 and human epidermal growth factor receptor 2 (HER2) status. The ErbB2/HER2 gene is a proto-oncogene located in the long arm of chromosome 17 (17q21–q22), implicated in the production of the transmembrane protein HER2, a membrane receptor protein of the tyrosine kinase type, located on the outer cell surface and leading to cell growth and differentiation (9). According to the College of American Pathologists (CAP), these are the only validated biomarkers to predict therapy response, together with patient age, histological grade, histological subtypes and TNM status [tumor size (T), lymph node status (N), and presence of distant metastases (M)] (7).

Indeed, their evaluation is also fundamental for subtype classification. Among different classifications, the most widely used one is the intrinsic classification by Perou and Sorlie, which identifies four subtypes of BC, based on a 50-gene expression signature (PAM50): luminal A and luminal B (expressing ER), basal-like, and HER2-enriched (without ER expression) (10). The first subtype, Luminal A BC, is characterized by ER+ and/or PR+, and HER2- configuration. It accounts for ~50–60% of BC cases and is associated with a comparably good prognosis. The second subtype, Luminal B BC, is characterized by an ER+ and/or PR+ (but lower than Luminal A), and HER2+ (Luminal B-like HER2+) or HER2- (Luminal B-like HER2-) arrangement. It accounts for ~30% of cases and may be associated with a higher Ki-67 value and a poorer prognosis (11). Approximately 10% of BC cases are represented by the HER2-enriched subtype, which is characterized by ER-, PR-, and HER2+ setting and an even poorer prognosis (12). The fourth subtype of BC is represented by basal-like/triple-negative breast cancer (TNBC), characterized by an ER-, PR-, and HER2- arrangement. This subtype, often occurring in younger women, constitutes ~15–20% of BC and is associated with a higher aggressiveness and a worse prognosis than the other molecular subtypes of BC (13).

**Figure 1:**
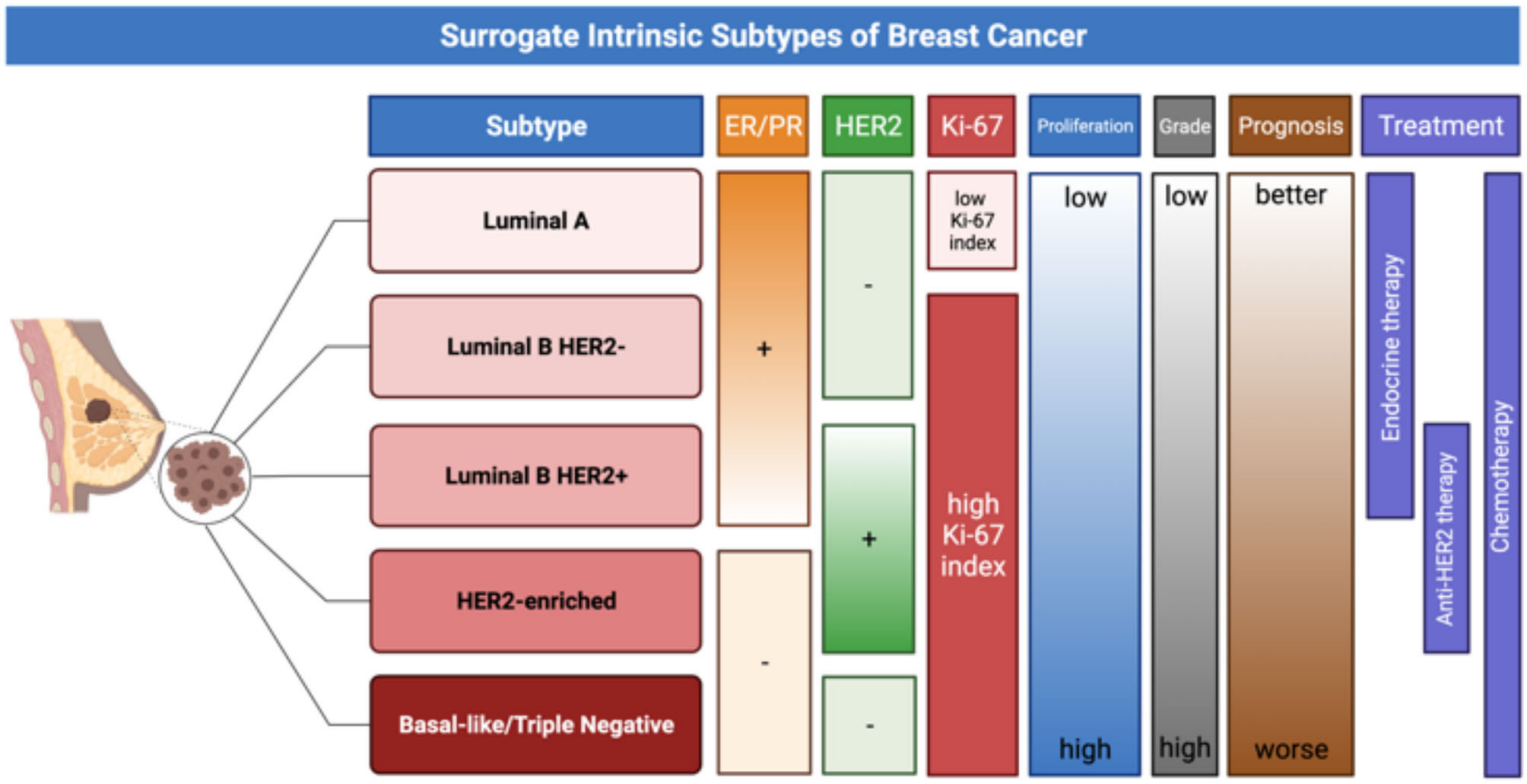
The figure provides a schematic representation of surrogate intrinsic BC subtypes (14), based on histologic and molecular features, which have significant implication for prognosis and treatment choice.

Currently, a classification of five surrogate intrinsic subtypes is more commonly used in clinical practice; this classification is based on the assessment of ER, PR, HER2, and the proliferation marker Ki-67 (14). The histologic and molecular features of breast cancer are summarized in Figure 1.

The treatment choice depends on the molecular and histological characteristics of the tumor and therefore it is important to consider these biomarkers for their therapeutic implications. In particular, HER2 protein overexpression represents a negative prognostic factor, being more commonly found in cases of high-grade tumors and in the presence of lymph node metastases. It is associated with a high mortality rate (9). Moreover, it has been demonstrated that hormone receptor positive tumors show a response to hormone therapy and usually have a more indolent course: hormone treatment often represents the first-line therapy in metastatic BC expressing hormone receptors (2, 8, 11). However, there are fundamental limitations for proper patient assessment. First, it is not always possible to identify the receptor status of the disease, especially in metastatic patients with lesions difficult to biopsy. Second, it must be remembered that the receptor status of secondary lesions may be different from the one of the primary tumor. Third, an invasive assessment of the receptor status for every new appearing lesion is not feasible.

In recent years, nuclear medical imaging (particularly receptor-specific imaging) has played an increasingly important and intriguing role through the development of new probes, capable of overcoming the limitations of *in-vitro* assessment (based on invasive tests and multiple biopsies), non-invasively obtaining different tumor characteristics *in-vivo*, which are crucial for optimal and personalized treatment.

**Figure 2:**
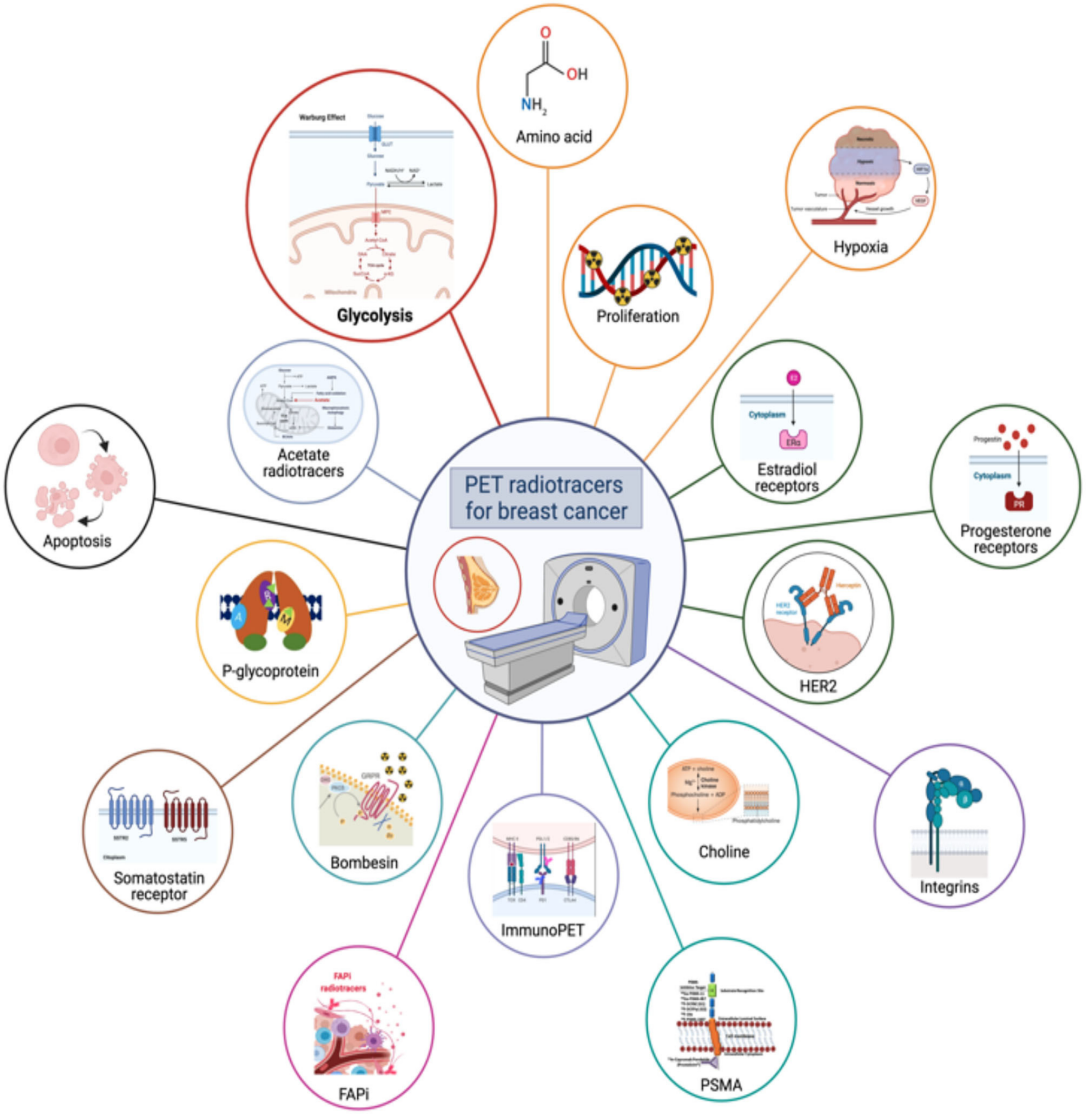
The figure shows a schematic representation of all major radiotracers currently in clinical use in BC or under evaluation.

Here, we will review several PET probes already used in clinical practice for BC and several promising radiopharmaceuticals currently under investigation for further use in this field (as shown in Figure 2), with the aim of analyzing their advantages and limitations and possible future developments.

## Materials and Methods

We searched the PubMed, PMC, Scopus, Google Scholar, Embase, Web of Science, and Cochrane library databases (between January 1990 and December 2021), using the following both as text and as MeSH terms: (“breast neoplasm^*^” OR “breast” OR “breast cancer^*^”) AND (“PET” OR “positron emission tomography” OR “PET/CT” OR “PET/MR”) AND (“FDG” OR “MET” OR “FLT” OR “FMISO” OR “FES” OR “estradiol” OR “trastuzumab” OR “PD-L1” OR “PSMA” OR “FAPI” OR “FACBC” OR “flucicovine” OR “FAZA” OR “GRPR” OR “DOTATOC” OR “DOTATATE”). No language restriction was applied to the search, but only articles in English were reviewed.

The systematic literature search returned 1,123 articles. Additional filters, such as original article and/or research article, and study including only humans and with 10 or more subjects, were used. Reviews, clinical reports, meeting abstracts, and editorial comments were excluded. Due to the comparably high number of studies available on this topic, an additional exclusion was based on citation threshold >20 citations for years 2010–2019 and >10 citation for 2020. According to the PRISMA flow-chart (15), after duplicate removal, 175 articles have been considered, fully read, analyzed, and extensively described according to their title and abstract. Relevant studies that were not obtained by the original search were included through cross-references; most relevant reviews on this topic were also reported.

## Molecular Imaging of Glycolysis

### 2-[^18^F]-Fluoro-2-Deoxy-D-Glucose ([^18^F]F-FDG)

To date, 2-[^18^F]-fluoro-2-deoxy-D-glucose ([^18^F]F-FDG) represents the most widely used radiopharmaceutical for PET imaging of BC patients. Glucose metabolism in BC cells is increased compared with normal tissues due to increased glycolysis, a phenomenon known as Warburg effect. [^18^F]F-FDG is an excellent biomarker of metabolism resembling glucose, which is entering into neoplastic cells, characterized by an increased number of glucose membrane transporters (GLUTs). Once intra-cellularly, [^18^F]F-FDG is then phosphorylated by hexokinase into [^18^F]F-FDG-6-phosphate, but unlike glucose, [^18^F]F-FDG-6-phosphate is very slowly metabolized further and thus remains trapped in the cell (16).

Current evidence does not support the use of [^18^F]F-FDG PET/CT in the staging of locoregional-limited disease, although it may be useful when conventional methods are inconclusive (3). In contrast, [^18^F]F-FDG PET/CT is recommended for initial staging in patients with clinical stage ≥ IIB BC (preferably performed before surgery), and it may also be used for staging patients with clinical stage IIA BC (T1N1 or T2N0), again preferably performed before surgery. [^18^F]F-FDG PET/CT is also recommended in cases of suspected or known BC recurrence, for early assessment of response to neoadjuvant therapy (particularly in TNBC or HER2+ disease) and for assessment of response to systemic treatment of metastatic BC (particularly for bone metastases) (4, 5). Indeed, [^18^F]F-FDG PET/(CT or MRI) has shown high sensitivity for detecting locoregional and distant metastases both in staging and restaging setting, showing high prognostic value. A recent systematic review and meta-analysis by Han et al. (17) showed that [^18^F]F-FDG PET/CT significantly changes the initial staging in newly diagnosed BC patients, with a relevant impact on patient management: the pooled proportions of changes in stage and management were 25% [95% confidence interval (CI), 21–30%] and 18% (95% CI, 14–23%), respectively.

**Figure 3:**
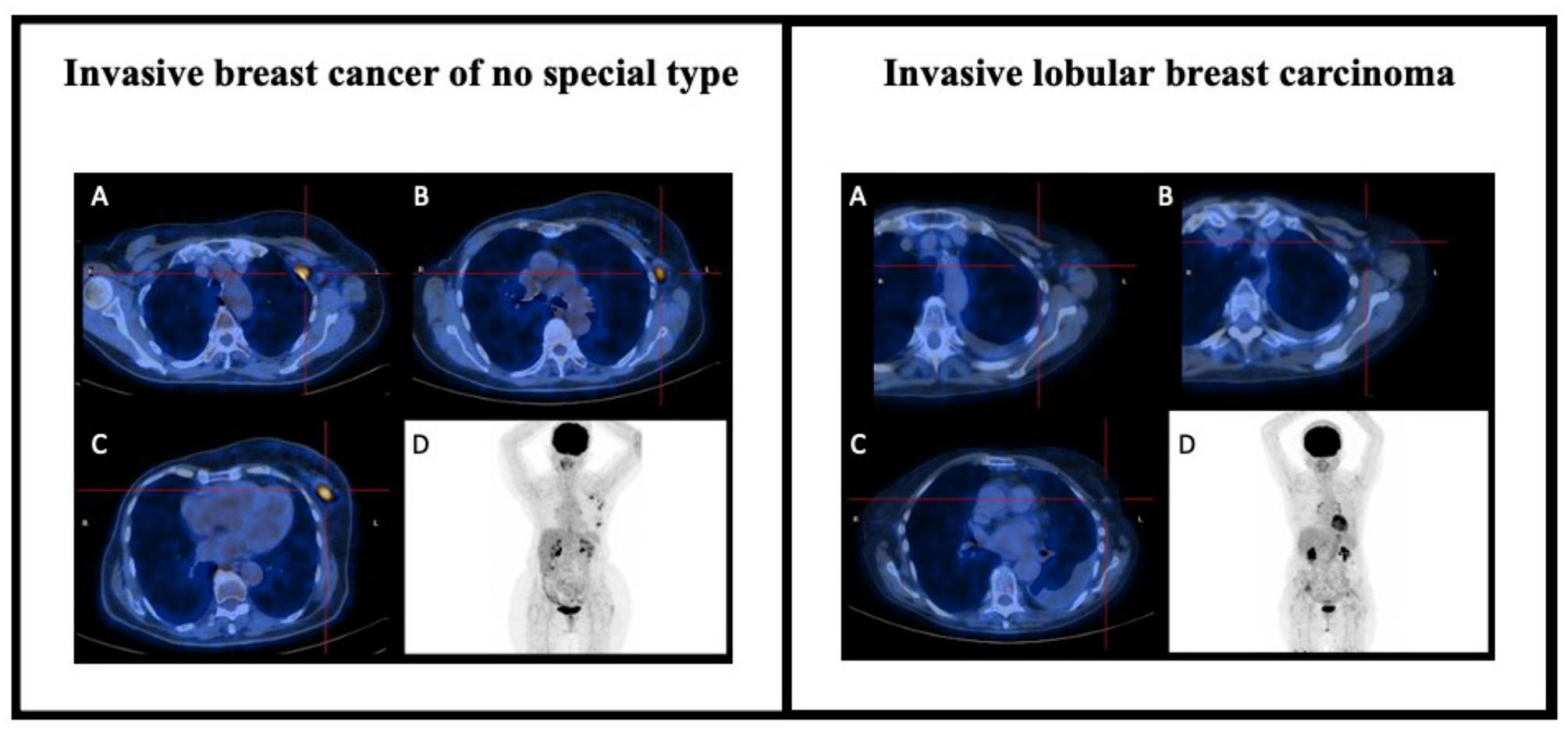
[^18^F]F-FDG PET/CT in BC staging. On the left, the case of an 83-year-old lady with previous *in situ* BC on the right and suspected recurrence of breast cancer on the left. Staging [^18^F]F-FDG PET/CT scan showed focal and elevated uptake at the level of the breast lesion on the left upper-external quadrant **(C)** and at the level of several ipsilateral axillary lymph nodes suspected for secondary localization of disease **(A,B)**, as also shown in the MIP images **(D)**. The cytologic finding was consistent with the clinical suspicion of IBC-NST recurrence (ER 98%, PR 99%, anti-Ki67 23%, anti c-erbB2 negative). On the right, the case of a 72-year-old lady hospitalized for dyspnea and CT findings of right pleural effusion concomitant to left mammary nodule and mediastinal lymphadenopathies. Staging PET scans did not show any FDG uptake in the suspicious lesions reported on CT scan, either in the breast **(C)** or in the ipsilateral axillary lymph nodes **(A,B)**, as also shown in MIP images **(D)**. The cytological findings were consistent with the clinical suspicion of ILBC (ER 78%, PR 55%, anti-Ki67 46%, anti c-erbB2 negative).

However, it is necessary to account for potential limitations of [^18^F]F-FDG PET/CT examinations in order to provide an appropriate interpretation: from the small tumor size (because of the low spatial resolution of PET tomographs and the partial volume effect) to the low sensitivity for specific histological subtypes with low [^18^F]F-FDG avidity. Invasive ductal carcinoma (IDC) shows higher [^18^F]F-FDG uptake than both ILBC and ductal carcinoma *in situ* (DCIS), as shown in Figure 3, making [^18^F]F-FDG PET/CT more effective in staging invasive ductal carcinoma (18).

[^18^F]F-FDG uptake may also vary depending on the receptor status of a lesion: lower uptake is observed in well-differentiated tumors (ER-positive and PR-positive) compared to ER-negative and PR-negative tumors. Several studies have shown that the standardized uptake value (SUV) is substantially higher in the TNBC subtype. Among luminal tumors, however, [^18^F]F-FDG uptake is lower in luminal A than in luminal B subtype (19–21). In general, higher [^18^F]F-FDG uptake is associated with a worse prognosis, as in cases of grade 3 tumors compared to grade 1 and 2 tumors (according to the Elston-Ellis modification of the Scarff-Bloom-Richardson (SBR) classification system), in tumors with increased proliferation (as assessed by the Ki67 index) and in tumors with mutated p53 (22). It should be remembered that [^18^F]F-FDG is not a specific radiotracer for BC or malignancy in general, and numerous conditions may lead to false-positive results, such as infection, fibroadenoma, ductal adenoma, inflammatory granulomatous mastitis, and fibrocystic changes (23–28). Some authors investigated the use of late imaging to improve specificity, since uptake usually increases on delayed images in case of malignancy, while it often decreases in inflammatory lesions (29, 30). However, [^18^F]F-FDG can only provide information on cellular metabolism and not on other tumor characteristics, such as proliferation and receptor status. Hence several other radiopharmaceuticals have been developed with the aim to assess other more specific features of BC lesions, overcoming the limitations of [^18^F]F-FDG.

## Molecular Imaging of Amino Acid Transporter, Cellular Proliferation and Hypoxia

### Amino Acid Transporter

In BC, as well as in other malignancies, increased protein synthesis has been observed associated with increased amino acid consumption and overexpression of amino acid transporters in the cell membrane (31).

L-methyl-[^11^C]-methionine ([^11^C]C-MET) represents one of the first radiolabeled amino acids used for the assessment of amino acid metabolism in oncologic PET imaging (32), in one of the first studies on the use of [^11^C]C-MET in BC patients by Leskinen-Kallio et al., both the primary tumor and metastases could be visualized, with a correlation between [^11^C]C-MET uptake and the fraction of cells in mitosis (S phase) in these lesions, indicating that [^11^C]C-MET uptake may be related to the proliferation rate of BC.

Subsequent studies focused on assessing treatment response in BC, demonstrating that [^11^C]C-MET uptake was reduced in responsive lesions, while it was unchanged or even increased in patients with progressing disease (33, 34). In particular, a reduction in [^11^C]C-MET uptake at the level of BC lesions can also occur early after the first treatment cycle (chemotherapy or hormone therapy), before objective clinical response or reduction in lesion size, thereby potentially allowing for an early discrimination between responding and non-responding patients (35).

**Figure 4:**
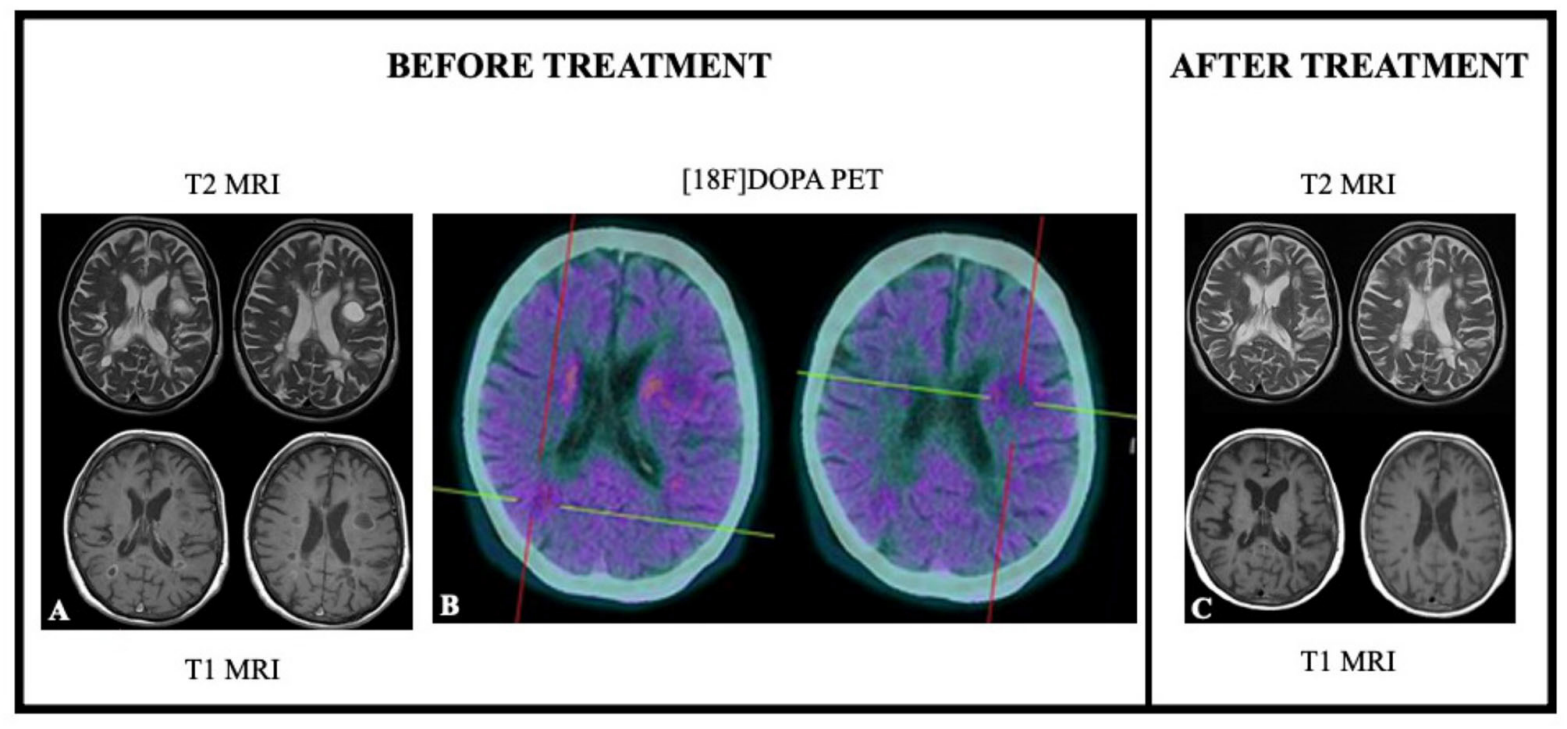
[^18^F]F-DOPA PET and MRI results in a case of differential diagnosis between tumefactive plaque due to multiple sclerosis and brain metastasis from breast cancer. Seventy-one years old female patient previously treated for BC. ceMRI showed the presence of at least two hyperintense at T2 sequences **(A)** and hypointense at T1 sequences **(A)** (the largest in left frontal lobe). [^18^F]F-DOPA PET/CT showed a very mild tracer uptake supporting the hypothesis of tumefactive lesion due to multiple sclerosis **(B)**. ceMRI performed 3 months after therapy administration showed reduction in both lesion size and contrast enhancement **(C)**.

In the literature, few studies with small sample size directly compared [^18^F]F-FDG PET/CT and [^11^C]C-MET PET/CT treatment response assessment in BC patients. Although [^11^C]C-MET was favored in some individual cases (36), in the majority of cases substantially overlapping results in terms of efficacy were obtained with these two radiotracers, both showing low diagnostic value in small lesions (37). [^11^C]C-MET and other amino acid radiotracers, such as 6-[^18^F]F-fluoro-l-dopa ([^18^F]-FDOPA), are particularly useful for the detection of brain metastases, to distinguish recurrent or progressive brain metastases (RPBM) from late or delayed radiation injury (LDRI) and also for the differential diagnosis with other pseudo-tumoral lesions, such as tumefactive multiple sclerosis (TMS) or brain abscesses, as demonstrated in the Figure 4 (38–40).

The main limitations of [^11^C]C-MET are: (1) a potential suboptimal evaluation of metastatic lesions in the liver and bone marrow, due to the high physiological tracer uptake in these organs (2, 41) the short half-life of only 20 min of [^11^C], limiting its use to centers with an on-site cyclotron; (3) the presence of non-protein metabolites, that may reduce the image quality.

Because of these limitations, other [^18^F]-labeled amino acids have been developed. Anti-1-amino-3-[^18^F]-fluorocyclo-butane-1-carboxylic acid ([^18^F]-fluciclovine or [^18^F]F-FACBC) is a synthetic amino acid, a leucine analog, mainly used in patients with prostate cancer, particularly in cases of biochemical disease recurrence (42–44).

Studies have shown that uptake of [^18^F]F-FACBC is higher in BC than in benign lesions and healthy breast tissue, with higher uptake in patients with higher tumor grade. This radiotracer is also suitable for the detection of metastasis (such as bone, lung, brain, and lymph node metastases). Interestingly, two different studies showed that [^18^F]F-FACBC avidity is higher than [^18^F]F-FDG avidity in ILBC and equal to [^18^F]F-FDG avidity in IDC (45, 46). Furthermore, Ulaner et al. observed not only that primary ILCs (4/14) have higher [^18^F]F-FACBC avidity than [^18^F]F-FDG avidity (median SUVmax 6.1 vs. 3.7, respectively), but also that primary IDCs have an inverse relationship, with lower [^18^F]F-FACBC avidity than [^18^F]FDG avidity (median SUVmax 6.8 vs. 10.0, respectively) (45).

[^18^F]F-FACBC PET/CT has also been used to assess response to neoadjuvant chemotherapy in patients with advanced BC. Ulaner et al. observed that changes in [^18^F]F-FACBC uptake correlated with pathological tumor response (47). However, [^18^F]F-FACBC is characterized by physiological high hepatic uptake and this represents a limitation for the detection of secondary lesions in the liver, a common site of BC metastases (46).

Hence, other amino acid metabolism radiotracers were developed. Two different radiotracers address the ${\text{x}}_{\text{c}}^{-}$ transporter, which mediates cellular uptake of cysteine by exchanging glutamate. The radiotracer (4S)-4-(3-[^18^F]-fluoropropyl)-l-glutamate ([^18^F]F-FSPG) was evaluated by Baek et al. in 5 BC patients. They demonstrated that [^18^F]F-FSPG uptake may vary between different histological or molecular subtypes, however, detecting only 30 of 73 BC lesions with known [^18^F]F-FDG avidity (48). Similar results were also obtained by studies on [^18^F]-5-fluoro-aminosuberic acid ([^18^F]F-FASu), which might have higher sensitivity than [^18^F]F-FDG for some tumor subtypes of BC (49, 50). However, the use of both tracers today is limited to a research setting.

Finally, other two interesting radiotracers are [^11^C]C-labeled tyrosine (L-[1-^11^C]C-tyrosine), which appears to be more accurate than [^18^F]F-FDG in differentiating malignant from benign lesions (51), and [^18^F]-(2S, 4R)4-fluoroglutamine ([^18^F]F-FGln), which has been recently used in the assessment of glutamine pool changes in patients with TNBC (52).

### Cellular Proliferation

One of the characteristic biological feature of malignant tumors is their increase in cell proliferation, which is correlated with tumor aggressiveness (53).

Thymidine is the only nucleotide incorporated into DNA but not RNA, making it an attractive target for the development of novel radiotracers that may allow for an assessment of DNA synthesis through the thymidine salvage pathway (54). One of the first cell proliferation probes developed was [^11^C]C-thymidine, which was rapidly discarded in favor of new [^18^F]-labeled probes due to the short half-life of [^11^C], demanding radiochemistry, and complicated pattern analysis (55–59).

Second-generation [^18^F]-labeled probes have been developed to overcome these limitations. Today, the most widely one used is [^18^F]-fluoro-3′-deoxy-3′-L-fluorothymidine ([^18^F]F-FLT). [^18^F]F-FLT enters cells by passive diffusion and *via* the equilibrative nucleoside transporter 1 (ENT1), and is then phosphorylated by thymidine kinase-1 (TK-1), but cannot participate further in DNA synthesis and remains trapped intracellularly. Although this radiopharmaceutical is not used in clinical routine, some studies have shown that [^18^F]F-FLT can detect BC lesions, both primary and secondary ones (54, 60). In particular, a strong correlation between [^18^F]F-FLT uptake (in terms of SUV) and the standard immunohistochemical marker of proliferation, Ki-67, has been observed (61, 62).

Despite this correlation, there are some limitations that need to be taken into account, such as the lower uptake gradient of [^18^F]F-FLT compared to [^18^F]F-FDG into BC lesions (with potential false negative results) and its high uptake into liver and bone marrow (with potential disadvantages for lesion detection in these organs) (54, 63). Although [^18^F]F-FLT is absorbed to a lesser extent than [^18^F]F-FDG in inflammatory tissue, leading to less false positives (64), the use of [^18^F]F-FLT PET/CT for staging has been discouraged by these issues.

Further studies have also evaluated the role of this radiotracer in assessing treatment response. Pio et al. showed that [^18^F]F-FLT uptake could predict changes in tumor proliferation after a cycle of cytotoxic chemotherapy (65). Kenny et al. observed an early reduction of [^18^F]F-FLT uptake into BC lesions, as an expression of change in cell proliferation, as early as 1 week after chemotherapy, and before an appreciable reduction in lesion size on CT (66). In 2021, López-Vega et al. evaluated, in a prospective phase II study, the accuracy of [^18^F]F-FLT PET/CT for the detection of proliferative status in 70 patients with primary stage II/III BC at baseline, during bevacizumab treatment (cycle 1; C1), and after four cycles of neoadjuvant docetaxel doxorubicin and bevacizumab every 3 weeks (C2–C5). They observed a significant decrease in tumor proliferation as measured by [^18^F]F-FLT uptake during C1 (*p* ≤ 0.001) compared to baseline, independent of tumor subtype (67).

However, in two recent different studies, [^18^F]F-FLT PET/CT did not show an advantage over [^18^F]F-FDG PET/CT in predicting treatment response and survival in patients with metastatic BC. In one study, Romine et al. evaluated the ability of these two radiotracers to measure early response to endocrine therapy from baseline until surgical resection in two separate cohorts of women with early stage ER+ BC: 22 patients underwent two sequential [^18^F]F-FDG scans and 27 patients underwent two sequential [^18^F]F-FLT scans, thereof the first scan prior to endocrine therapy and the second one pre-operatively. Pre- and post-therapy PET measures showed strong rank-order agreement with Ki-67 percentages for both radiotracers, demonstrating no concrete advantage in using [^18^F]F-FLT instead of [^18^F]F-FDG (68). In another prospective study, Su et al. aimed to compare the value of interim [^18^F]F-FLT and [^18^F]F-FDG PET/CT to predict treatment outcome in 25 patients with metastatic BC after salvage therapy. All patients had dual radiotracer PET/CT at baseline, and after the 1st and 2nd cycle of systemic chemotherapy. Metabolic response determined by Response Criteria in Solid Tumors (PERCIST) on interim [^18^F]F-FDG PET/CT after 2 cycles showed a high accuracy in predicting clinical response (AUC = 0.801, *p* = 0.011), with significantly higher 2-year progression free survival (PFS) for responders (53.8 vs. 16.7%, respectively, *p* = 0.014) and higher 2-year overall survival (OS) (100 vs. 47.6%, respectively, *p* = 0.046) compared with non-responders. In contrast, survival differences between responders and non-responders at interim [^18^F]F-FLT PET/CT were not significant (69).

The 1-(29-deoxy-29-fluoro-b-D-arabinofuranosyl) thymine ([^18^F]F-FMAU) is another [^18^F]-labeled thymidine analog used for PET imaging. Also for this new probe, the few available studies have low patient numbers. Despite this limitation, Sun et al. observed a good tumor to healthy tissue ratio (average BC SUVmax of 2.17), low uptake at the bone marrow level, but high physiological uptake at the hepatic level (70). Moreover, a 5–10 times lower uptake of [^18^F]F-FMAU was observed in more aggressive tumors, such as TNBC, compared to [^18^F]F-FLT, probably because [^18^F]F-FMAU is a substrate of the mitochondrial enzyme thymidine kinase-2 (TK-2) with low specificity for TK-1 (71).

New proliferation probes, such as [^18^F]-benzamide analogs, that bind to sigma 2 (s2) receptors, are under development. The function of these receptors appears to be associated with the potassium and calcium ion channel transport (72). From this radiotracer category, the N-(4-(6,7-dimethoxy-3,4-dihydroisoquinoline-2(1H)-yl)butyl)-2-(2-[^18^F]-fluoroethoxy)-5-methylbenzamide ([^18^F]F-ISO-1) was the first one to be evaluated in a clinical study with 30 cancer patients (13/30 with proven BC). The study observed a correlation between tumor uptake and ki-67. However, also this radiotracer showed high uptake in the liver and pancreas, which may limit its use in BC patients (73). Finally, McDonald et al. reported the results of a prospective clinical trial (NCT02284919) dedicated to [^18^F]F-ISO-1 PET/CT in 28 women with 29 primary invasive BC. Tumors stratified into the high Ki-67 group (≥20%) had higher SUVmax than the low Ki-67 group (<20%) (*p* = 0.02). SUVmax showed a positive correlation with Ki-67 in all breast cancer subtypes (ρ = 0.46, *p* = 0.01) and SUVmax corrected for partial volume was positively correlated with Ki-67 in invasive ductal carcinoma (ρ = 0.51, *p* = 0.02). The study also showed that the uptake of [^18^F]F-ISO-1 in breast cancer correlates modestly with the *in-vitro* Ki-67, obtained by biopsy (74).

### Hypoxia

Hypoxia in cancer lesions occurs when there is an imbalance between increased cellular metabolism and insufficient oxygen (O2) supply; characterized by high O2 consumption, a disorganized tumor vasculature with slow blood flow and consequent low arteriolar supply to cancer cells. These phenomena are associated with an overexpression of hypoxia-inducible factor 1 (HIF)-1 and increased glycolysis, angiogenesis and resistance to apoptosis (75).

The presence of hypoxia in many tumor types, including BC, has been shown to induce resistance to both chemotherapy and radiation therapy, representing a negative prognostic factor (76).

Radiolabeled nitroimidazoles represent the most widely developed radiopharmaceuticals for PET imaging of hypoxia in oncology. Among these, [^18^F]fluoromisonidazole ([^18^F]F-FMISO) is the one most widely used (77). Owing to its lipophilic nature, it diffuses through cell membranes, is then reduced by nitroreductases in vital cells (when tissue pO2 is <10 mmHg) and remains trapped within the cells. At 2–4 h after administration, retention is considered specific for cellular hypoxia (78).

Although many with [^18^F]F-FMISO PET/CT studies have been conducted in other types of cancer (79), only a few studies covered [^18^F]F-FMISO PET/CT in BC. In a study of Rajendran et al. on four different tumor types, including seven BC patients, glucose metabolism ([^18^F]F-FDG PET/CT) and hypoxia ([^18^F]F-FMISO PET/CT) were directly compared. They reported a discordance between the uptake of these two radiotracers, which might be tumor-type-specific: the mean correlation coefficients between [^18^F]F-FMISO and [^18^F]F-FDG SUV-uptake were 0.47 for BC. The linear association between [^18^F]F-FMISO and [^18^F]F-FDG varied among the tumor types examined, with the order sarcoma < brain < breast < head and neck. The differences between average correlations within the tumor types were highly significant (*p* < 0.005) (80). Chen et al. used [^18^F]F-FMISO in patients with ER-positive stage II-IV BC both before and after 3 months of aromatase inhibitor therapy, showing the ability of this method to predict resistance to endocrine therapy (81). In the aforementioned prospective phase II study by López-Vega et al., the 70 patients with primary stage II/III BC were evaluated not only with [^18^F]F-FLT, but also with [^18^F]F-FMISO PET/CT during bevacizumab treatment. Interestingly, [^18^F]FMISO SUVmax at baseline was modestly correlated with VEGFR-2 expression (ρ = 0.26, *p* = 0.02), even if [^18^F]F-FMISO uptake did not differ significantly before or after bevacizumab therapy or by BC subtype (67).

The main disadvantages of [^18^F]F-FMISO are a slow clearance from blood and healthy tissue (resulting in a reduced target/background ratio), its relatively short half-life (110 min), and the need for imaging acquisitions at 2–3 h after administration. Therefore, second-generation compounds have been developed that offer a better target/background ratio and have better pharmacokinetic properties. Among these new probes is [^18^F]-fluoroazomycin-arabinoside ([^18^F]F-FAZA), which is more hydrophilic than [^18^F]F-FMISO and shows better clearance kinetics, with a more favorable target/background ratio (82).

Other hypoxia probes for PET imaging already studied in varies tumor type, but not yet in BC, include: -the more lipophilic [^18^F]-2-nitroimidazol-pentafluoropropyl acetamide (83); -the non-nitroimidazole compound copper(II)diacetyl-bisN(4)-methylthiosemicarbazone (Cu-ATSM), which can be labeled with either [^60^Cu] or [^64^Cu] (84–87); -the [^18^F]-fluoroerythronitroimidazole ([^18^F]F-FETNIM) (88); -the [^18^F]-1-(2-1-(1H-methyl)ethoxy)-methyl-2nitroimidazole ([^18^F]F-RP-170) (89); -the [^18^F]-3-fluoro-2-(4-((2-nitro-1H-imidazol-1-yl)methyl)-1H-1,2,3-triazol-1-yl)propan-1-ol ([^18^F]F-HX4) (90).

## Molecular Imaging of Receptors

### Estradiol Receptor

Being aware of the receptor status of BC lesions is essential not only for a correct characterization of the disease from an anatomical and pathological point of view, but also for defining eligibility for endocrine therapy targeting steroid receptors, which represents one of the most effective systemic treatments. As already mentioned, the histological and molecular classification of BC is mainly based on the expression of ER, PR, HER2, and the proliferation marker Ki-67. ER- or PR-positive BCs are usually characterized by lower aggressiveness and better prognosis than HER2-enriched and triple-negative subtypes (12, 13). However, the receptor status of the disease is not always easily identifiable, especially in metastatic patients where secondary lesions may have receptor status different to the primary tumor (91).

[^18^F]-labeled receptor ligands have gained importance over the years, allowing for an *in-vivo* assessment of the receptor status both in the primary tumor and in metastatic lesions, such as [^18^F]-labeled estradiol (especially endogenous estradiol, E2) for ER imaging (especially ERα) (92, 93).

**Figure 5:**
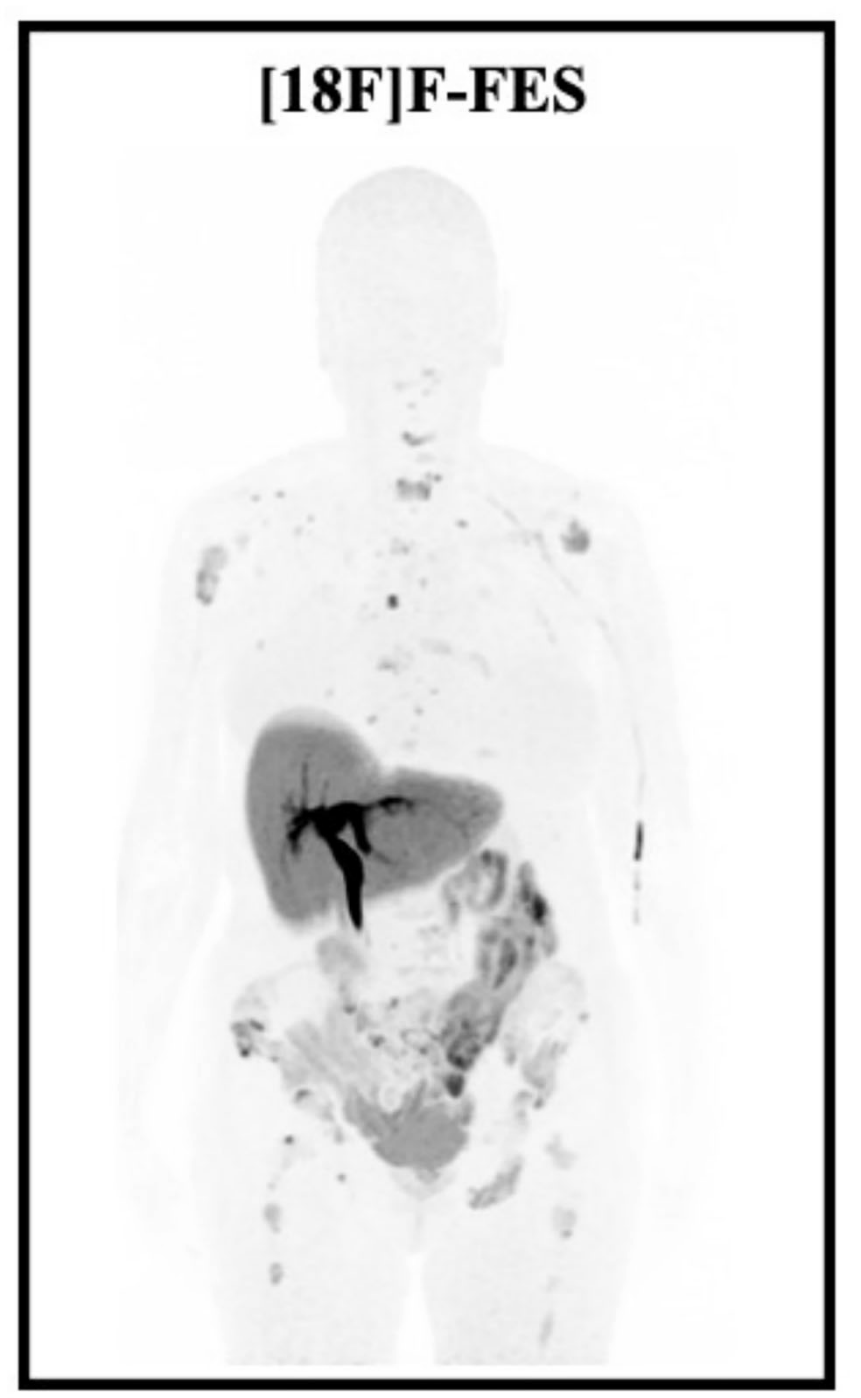
Low intensity MIP images showing the normal biodistribution of [^18^F]F-FES and multiple bone recurrence metastases of an ILBC, occured 5 years after surgery (SBRII, 15 mm, ER 80%, PR 80%, Ki67 40%, HER2 negative), radiotherapy and hormonotherapy with letrozole.

16α-[^18^F]-fluoro-17β-estradiol ([^18^F]F-FES) is an analog of E2, with an affinity for ERα slightly higher than that of E2 (94), and with a similar biodistribution. It is metabolized in the liver and excreted through the biliary tract and then reabsorbed through the small intestine (it does not accumulate physiologically in the large intestine) and is then eliminated mainly through the urine (only 5% in the feces) (95–97). An example of [^18^F]F-FES PET/CT scan is shown in Figure 5.

[^18^F]F-FES represents one of the clinically most widely used radiopharmaceuticals of this category: a high correlation between [^18^F]F-FES uptake and estrogen receptor concentration has been demonstrated already in 1980, confirmed on histopathological analysis after tumor excision (98). A recent 2020 meta-analysis by Kurland et al. (99) demonstrated that [^18^F]F-FES non-invasively characterizes ER ligand binding function in BC lesions with a sensitivity of 0.81 (0.73–0.87) and a specificity of 0.86 (0.68–0.94) compared to the histological standard of reference.

[^18^F]F-FES PET/CT examinations do not require any specific preparation, however—mainly as a measure of standardization—fasting is recommended and some foods, such as chocolate, should be avoided (100). Nevertheless, some pharmacological aspects have to be taken into account: in case of staging, treatment with ER antagonists (e.g., tamoxifen or fulvestrant) should be discontinued for ≥5 weeks before the examination. Aromatase inhibitors and luteinizing hormone-releasing hormone (LHRH) agonists can be continued (101).

Nowadays, [^18^F]F-FES PET/CT represents the main non-invasive method for the assessment of ER expression throughout the body, with important therapeutic implications: a low uptake of [^18^F]F-FES is associated with a higher failure rate of antihormonal treatment (101).

Although current guidelines do not recommend [^18^F]F-FES PET/CT as a diagnostic tool in patients with ER-positive BC, this radiotracer could be beneficial when conventional and [^18^F]F-FDG imaging is inconclusive, providing greater specificity than [^18^F]F-FDG PET/CT. In 2019, Liu et al. (102) investigated whether and how [^18^F]FES-PET/CT affects the management of 19 newly diagnosed estrogen receptor positive BC patients. A total of 238 lesions was analyzed, there of 216 were detected by [^18^F]F-FES and 197 by [^18^F]F-FDG PET/CT, resulting in a sensitivity of 90.8% for [^18^F]F-FES vs. 82.8% for [^18^F]F-FDG PET/CT, corroborated by CT and/or other imaging. The application of [^18^F]F-FES in addition to [^18^F]F-FDG PET/CT changed the management in 5 of 19 patients (26.3%), highlighting lesions that were negative or equivocal on [^18^F]F-FDG. Moreover, [^18^F]F-FDG PET/CT demonstrates lower sensitivity for ILC, which is almost always (95%) ER-positive. Therefore, Ulaner et al. (103) evaluated 7 metastatic ILC patients with synchronous [^18^F]F-FDG PET/CT and [^18^F]F-FES PET/CT. In this cohort, 254 lesions suggestive of malignancy were detected by increased [^18^F]F-FES uptake (SUVmax, 2.6-17.9), compared to 111 lesions detected by increased [^18^F]FDG uptake (SUVmax, 3.3-9.9). Notably, [^18^F]F-FES PET/CT detected more metastatic lesions than [^18^F]F-FDG PET/CT in 5 of 7 patients (71%). All [^18^F]F-FDG-positive lesions were also [^18^F]F-FES-positive. Boers et al. (104) evaluated whether [^18^F]F-FES PET/CT could resolve the remaining physician's clinical dilemma in 83 BC patients with suspected heterogeneous ER expression. The dilemmas were as follows: inability to determine the extent of metastatic disease or suspected metastatic disease with standard work-up (*n* = 52), unclear ER status of the tumor (*n* = 31), and inability to determine which primary tumor was responsible for the metastases (*n* = 17). A total of 100 PET/CT scans of 83 patients were analyzed: dilemmas were resolved by [^18^F]F-FES PET/CT in 87 of 100 scans (87%). Furthermore, the frequency of resolved dilemmas was correlated with whether the scans were [^18^F]FES-positive (*n* = 63) or [^18^F]F-FES-negative (*n* = 37; *p* < 0.001); demonstrating the usefulness of this radiotracer in cases of clinical dilemma.

In addition, [^18^F]F-FES PET/CT was found useful in assessing the degree of response to antihormonal therapy in patients with metastatic BC. [^18^F]F-FES PET/CT can indicate whether the malignant lesions continue to express ER, and thus provide a rationale for continuing or switching lines of antihormonal therapy, or switching to another type of treatment with absent ER expression (105–108). Low or absent uptake of [^18^F]F-FES in BC lesions correlates with greater resistance to antihormonal therapy, and conversely, patients responding to endocrine therapy showed higher SUV (although no specific SUV thresholds exist to distinguish specific from non-specific uptake) (105, 106, 109). However, a meta-analysis of seven studies (in total 226 patients) by Evangelista et al. (110) showed that the role of [^18^F]F-FES PET/CT in predicting response to endocrine therapy in advanced BC still remains undetermined: pooled sensitivities and specificities were 63.9% (95% CI: 46.2–79.2%) vs. 66.7% (95% CI: 52.1–79.2%), and 28.6% (95% CI: 17.3–42.2%) vs. 62.1% (95% CI: 48.4–74.5%), for an SUV cutoff of 1.5 and 2.0, respectively.

[^18^F]F-FES PET/CT has also been used as a tool to predict response to neoadjuvant chemotherapy together with [^18^F]F-FDG PET/CT in BC patients. In a study of 18 patients, Yang et al. (111) have not found statistically different [^18^F]F-FDG SUVmax and tumor size among 10 responders and 8 non-responders. In contrast, lower SUVmax [^18^F]F-FES was found in responders (1.75 ± 0.66 vs. 4.42 ± 1.14; *p* = 0.002) and the [^18^F]F-FES/[^18^F]F-FDG SUVmax ratio also showed great value in predicting outcome (0.16 ± 0.06 vs. 0.54 ± 0.22; *p* = 0.002).

The main disadvantages of [^18^F]F-FES include high uptake in the liver, which may render the assessment of liver metastases difficult; rapid blood clearance, which may lead to lower tumoral uptake; and low selectivity for ERα and ERβ, resulting in reduced specificity of the procedure. In an attempt to overcome these limitations and make PET imaging more specific for ER expression, additional radiopharmaceuticals have been developed, such as 4-fluoro-11β-methoxy-16α-[^18^F]-fluoroestradiol (4FM-[^18^F]F-FES) (112) and 1-(2-(2-(2-[^18^F]fluoroethoxy)ethoxy)ethyl)-1H-1,2,3-triazole-estradiol ([^18^F]F-FETE) (113).

### Progesterone Receptor

Numerous physiologic functions are elicited by the progesterone receptor (PR) through progesterone binding, acting as a ligand-dependent transcription factor (114). The main target tissues are the ovaries, the uterus and mammary gland tissue (115). Progesterone represents a precursor molecule for the synthesis of estrogen, androgen, and adrenocortical steroids (116).

In ~50% of patients with estrogen-dependent BC, increased progesterone receptor expression is also observed. Therefore, the PR status can provide important prognostic and predictive information. In fact, PR is routinely assessed in clinical practice, since it predicts response to endocrine therapy (114).

In BC, the expression of ERα and PR are closely associated, because functioning ER can increase the expression of PR, which represents a surrogate biomarker for the presence of a functioning estrogenic pathway (117). In fact, ER-/PR+ cases are rare (< 1% of all BCs) (118). In case of ER+/PR+ BC, a response to endocrine treatment will probably be observed (in ~75%). In contrast, chances of response are lower in ER+/PR- cases (64). However, endocrine therapy still represents an option for these patients, which have an overall poor prognosis owing to their more aggressive disease than PR+ patients (119).

Based on these premises, the main advantage of PET imaging targeting PR is that it serves as a surrogate for ER expression, when this one is saturated by ongoing specific ER therapy.

21-Fluoro-16α-ethyl-19-norprogeserone ([^18^F]F-FENP) was one of the first [^18^F]-labeled ligands developed for PET imaging and showed a high affinity for PR, ~60-fold higher than progesterone, as observed by Pomper et al. (120). However, in clinical PET trials this ligand did not succeed, identifying only 50% of PR+ tumors (121). Owing to its high lipophilicity and metabolism, this radiotracer is characterized by high uptake into liver and adipose tissue, low target-to-background ratio for high background activity, and notable bone uptake for metabolic defluorination (121, 122).

Another radiotracer, [^18^F]F-FMNP, although characterized by high affinity and specificity for PR, it also showed high lipophilicity and an unfavorable metabolism as [^18^F]F-FENP, and hence has no clinical application (121).

6α-[^18^F]-Fluoroprogesterone showed the same limitations, characterized by high absorption in adipose tissue, relatively low target tissue selectivity and high bone absorption due to metabolic defluorination (123).

Among these PR radioligands, the most promising one that was also evaluated clinically is 21-^18^F-fluoro-16α,17α-[(R)-(1′-α-furylmethylidene)dioxy]-19-norpregn-4-ene-3,20-dione ([^18^F]F-FFNP), characterized by high PR binding affinity and low non-specific binding (124, 125). Dehdashti et al. demonstrated in their study how [^18^F]FFNP showed greater uptake in BC than in healthy breast tissue, and among BCs, greater uptake was observed in PR+ lesions than in PR- lesions (126). Moreover, [^18^F]F-FFNP is subject to minimal defluorination, resulting in low bone uptake, and it is less affected by hydrogenase metabolism, which unfavorably affects the biodistribution of the above-mentioned other PR radiotracers (121).

Fowler et al. used [^18^F]F-FFNP to predict response to endocrine therapy in a preclinical model of BC (127). Mammary cell lines (SSM1, SSM2, and SSM3) were implanted into mice and an imaging study was performed to determine whether changes in ERα/PR expression had predictive value for tumor response after endocrine therapy. In another preclinical study, they showed how the uptake of [^18^F]F-FFNP into the tumor increases after hormonal estrogen therapy, due to synergetic function between estrogen and progesterone receptors; but decreases after the end of therapy in responding lesions, as showed by Linden et al. (64).

In a prospective, phase 2, single-center, single-arm study (NCT02455453) published in 2021, Dehdashti et al. (128) demonstrated for the first time directly in humans the influence of estrogen on tumor progesterone receptors. Forty-three postmenopausal women with advanced ER+ BC underwent two [^18^F]F-FFNP PET/CT studies on 2 separate days, the second PET being performed after an estradiol challenge (a total dosage of 6 mg of estradiol) administered to detect an eventual “flare reaction” on the second PET. Interestingly, tumoral uptake of [^18^F]F-FFNP increased only in the 28 subjects with clinical benefit from estrogen therapy (responders), but not in the 15 without clinical benefit (no responders) (*p* < 0.0001), indicating 100% sensitivity and specificity. The authors also showed significantly longer survival (*p* < 0.0001) in responders, which renders this radiotracer highly predictive of response to estrogen therapy in women with ER+ BC, pioneering clinical trials in humans with this radiotracer.

### HER2

The use of radiolabeled monoclonal antibodies and derivatives for PET imaging is a field of great scientific interest. As mentioned above, HER2 is a member of the epidermal growth factor receptor (EGFR) family of tyrosine kinases, and is encoded by the HER2 proto-oncogene (9). HER2 is involved in a wide range of cellular processes, such as survival, proliferation, differentiation, maturation, metastatic spread, angiogenesis, invasion, and antiapoptotic functions (129).

HER2/erbB2 oncogene overexpression or amplification occurs in ~20% of BC patients, representing a negative prognostic factor, as HER2-positive BC is characterized by more aggressive tumor behavior (130, 131). Moreover, HER2 expression may vary between the primary tumor and metastatic lesions (intra-tumor or temporal heterogeneity), with discordant expression rates between 4 and 16% for HER2 expressions, affecting tumor behavior and response to treatment (132). Finally, HER expression may also change during treatment.

For these reasons, it is important to identify a non-invasive tool for monitoring HER2 expression levels *in vivo* during HER2-targeted therapy, particularly for the assessment of treatment response. As mentioned above for hormone receptors, HER2 PET/CT imaging with the development of new probes can also be a useful tool to determine HER2 expression and the location of HER2-positive tumor lesions in a non-invasive and total-body approach (133).

Trastuzumab (Herceptin) is a recombinant G1 immunoglobulin monoclonal antibody, targeting the extracellular domain of HER2, which is widely used in clinical medicine and hence represents an optimal target for the development of a new PET probe (134). [^89^Zr]Zr-trastuzumab is a PET imaging radiopharmaceutical capable of assessing HER2 expression in BC patients, both in primary and metastatic lesions, qualitatively and quantitatively. It may allow to improve the selection of patients who might benefit most from trastuzumab therapy (135, 136).

The first clinical study with [^89^Zr]Zr-trastuzumab was performed by Dijkers et al. (136) in 2010 with the aim to evaluate the biodistribution, the optimal dosage and time of radiotracer administration in 14 patients. PET imaging results clearly showed HER2-positive liver, bone, and brain lesions, with a great lesion to background ratio in the liver, spleen, kidneys, and brain. Owing to the long half-life of [^89^Zr] (78.4 h), imaging could still detect occult metastatic lesions 5 days after [^89^Zr]Zr-trastuzumab injection. On the other hand, this results in high “radiation exposure.” Subsequently, Laforest et al. demonstrated that optimal imaging requires at least 4 days between tracer injection and scanning, even if the liver was the dose-limiting organ for the correct visualization of liver metastasis at 4 days (137).

One of the first human study to evaluate the clinical impact of [^89^Zr]Zr-trastuzumab PET/CT was conducted by Gebhart et al. (138) in 2016, demonstrating in 56 BC patients that pretreatment imaging of HER2 targeting, combined with early metabolic ([^18^F]F-FDG) response assessment holds great promise to predict efficacy of HER2-targeting antibody-drug-conjugate trastuzumab emtansine (T-DM1). More recently, Bensch et al. (139) demonstrated that [^89^Zr]Zr-trastuzumab PET/CT supports clinical decision-making when HER2 status cannot be determined by biopsy.

The shorter half-life of [^64^Cu] (12.7 h) makes [^64^Cu]Cu-DOTA-trastuzumab a very attractive radiopharmaceutical for PET imaging. Tamura et al. evaluated PET imaging with [^64^Cu]Cu-DOTA trastuzumab in six HER2-positive BC patients, showing high activity in blood, but low activity in normal tissue, and a radiation exposure equal to that of [^18^F]F-FDG PET/CT (140). In addition, this radiopharmaceutical showed good capability to assess HER2 expression *in vivo*, distinguish HER2-positive from HER2-negative BC, and monitor changes in HER2 expression after therapeutic intervention. However, the high liver uptake reduces the ability of this radiotracer for assessing small lesions. This issue can be partially resolved by administering 45 mg of cold trastuzumab before PET to reduce hepatic uptake (141) or by performing the examination after a standard therapeutic dose of trastuzumab (142).

Another HER2 radiotracer, [^68^Ga]Ga-DOTA-ABY-002, has only been used for the detection of abdominal BC metastases (143). In 2016, Sorensen et al. (144) tested [^68^Ga]Ga-DOTA-ABY-025 in 16 patients with metastatic BC in a phase I/II study. Optimal whole-body PET images were obtained at 4 h after injection. Biopsies from 16 metastases in 12 patients were collected for verification of HER2 expression by immunohistochemistry and *in-situ* hybridization. PET SUV correlated with biopsy-derived HER2 scores (*r* = 0.91, *p* < 0.001) and uptake was five times higher in HER2-positive than in HER2-negative lesions with no overlap (*p* = 0.005). With regard to the dosimetry, the highest absorbed organ doses were seen in the kidneys, followed by the liver (145).

In order to overcome the problem of high background uptake and potential issues related to cross-calibration of scanning devices, Sandberg et al. (146) aimed to investigate the utility of a tumor-to-reference tissue-ratio (T/R) as a HER2 status discrimination strategy in 16 patients with HER2-positive/negative metastasized BC, scanned with [^68^Ga]Ga-ABY-025 PET/CT. Spleen was the best reference tissue and spleen-T/R was highly correlated to PET SUV in metastases after 2 h (*r* = 0.96, *p* < 0.001), reaching an accuracy of 100% for discriminating immunohistochemistry (IHC) HER2-positive and negative metastases at 4 h (PET) after injection.

Overall, clinical data on HER2 imaging in PET are still few and limited, but own great potential, particularly with encouraging preclinical data on the horizon.

## Integrins Targeted Radiotracers

The integrins are a family of transmembrane proteins involved in many fundamental cellular processes, such as interaction between the cell and the extracellular matrix, and mediation between cells. Moreover, the integrins influence extracellular and intracellular signaling pathways, including apoptosis, and play also a key role in tumor progression and metastasis (147). In particular, the αvβ3 subclass is involved in tumor transformation, angiogenesis, local invasiveness, and metastatic potential. It is known to be over-expressed by both angiogenic endothelium and cells in several tumor types, including BC (148). The αvβ3 subclass is involved among various angiogenic signaling cascades, such as the vascular endothelial growth factor (VEGF) pathway. The inhibition of VEGF significantly suppresses the expression of αvβ3 on tumor cells, with reduced microvascular density, thus it has been proposed as a marker of angiogenic activity (149). Bevacizumab (anti-VEGF antibody) represents one of the most widely used drugs for anti-angiogenic treatment, through inhibition of the VEGF pathway, in several tumor types, such as in non-small cell lung cancer, colorectal cancer, and glioma (150). Arginine-glycine-aspartic acid (RGD) peptide ligands have shown high affinity for αvβ3-integrin and can be radiolabeled for PET imaging of angiogenesis or tumor development (151, 152). In a preclinical study performed in mice with BC, scans were performed at baseline and after bevacizumab therapy. [^68^Ga]Ga-TRAP (RGD)3 uptake was reduced in mice treated with the VEGF antibody compared to the untreated group, where uptake did not change significantly (153). With the ability to non-invasively visualize αvβ3 expression by PET in BC patients, important data on the integrin levels in tumor lesions might be obtained with the goal select the most suitable patients for antiangiogenic treatment and to assess the degree of response to this type of therapy. In fact, in a clinical study of 16 patients with BC (154), it was observed that [^18^F]F-Galacto-RGD was absorbed by all tumor lesions, both primary and metastatic ones, although with heterogeneous uptake levels. [^68^Ga]Ga-NODAGA-THERANOST, a second-generation compound, was used in 2 patients, one of them with BC, showing promising data (155). [^18^F]F-fluciclatide ([^18^F]F-AH111585) appears to be more stable, safe, and well-tolerated (156, 157) providing a good target/background ratio. This probe allows detection of both primary and metastatic BC lesions, although evaluation of liver metastases may be suboptimal due to high physiological hepatic uptake (66). Even with [^18^F]F-fluciclatide, tumor uptake was observed to vary considerably between individuals and between tumor types, and even between tumors of the same type within a patient. In order to assess this heterogeneity, Tomasi et al. (158) employed a dynamic acquisition of [^18^F]F-fluciclatide PET data in BC patients with metastases. In 8 patients with BC [^18^F]F-FPPRGD2 was used to evaluate the expression of integrin avβ3. This probe is characterized by high specificity, although showing high uptake in the liver and kidneys, which may limit the evaluation of metastases in these organs (159). Gaykema et al. studied [^89^Zr]Zr-bevacizumab in 23 patients with BC undergoing PET imaging 4 days after administration, which is considered the optimal time-point and it is possible thanks to the long half-life of [^89^Zr] (78.4 h). They observed a significantly higher uptake in tumor lesions than in healthy tissue, and that luminal B tumors showed greater uptake compared to luminal A tumors. However, this radiotracer overall yielded low sensitivity in detecting lymph node lesions (160).

## Choline Analogs Radiotracers

Since many years, it has been observed—mainly through spectroscopy MRI studies—that in many tumor types there is an altered choline metabolism (161). Choline is an essential component of the cell membrane and a building block for the synthesis of fatty acids (162). When choline enters the cell, it is phosphorylated by choline kinase-α, turning into phosphocholine, which is trapped inside the cell. This phenomenon has been observed to increase with parathyroid adenoma mainly in primary hyperparathyroidism (163) and with malignant transformation in some tumor types, such as BC (particularly HER2 BC) (164). Although most clinical trials have been performed for prostate cancer (165), promising results have also been obtained with N-[^11^C]methyl-choline ([^11^C]C-choline) in BC patients. Increased uptake in BC compared to normal tissue was observed, which was related to the expression and activity of choline kinase-α since choline is required for membrane synthesis in actively proliferating cells (166). After several case reports, in 2009, Contractor et al. (167) first evaluated the use of [^11^C]C-choline PET/CT in in 32 individuals with primary or metastatic ER–positive BC. Their study demonstrated that breast tumors were well-visualized in 30 of 32 patients with good tumor background ratio. In 2010, Kenny et al. (168) demonstrated the reproducibility of the [^11^C]C-choline PET/CT uptake variables in 21 BC patients, performing a dual time point evaluation. They also demonstrated that trastuzumab therapy decreases [^11^C]C-choline uptake in BC lesions. One of the main disadvantages of this PET imaging method is the high physiological hepatic uptake, which may limit the evaluation of liver metastases. Only a few clinical studies have been performed on [^18^F]F-choline in BC patients, limited to case reports (169). More recently, Saad et al. (170) described the pathological distribution of [^18^F]F-choline PET/CT scan, acquired 40 min after injection, and pitfalls in 21 BC patients. All patients showed potentially false negative lesions, predominantly caused by physiological uptake in the liver, spleen, pancreas, bowel, axial skeleton (85–100%), inflammation and benign lesions (4.7%), and appendicular skeleton (4.7–19.0%). Also, [^18^F]F-choline uptake was higher in lesions of premenopausal women.

## Prostate-Specific Membrane Antigen Radiotracers

Prostate-specific membrane antigen (PSMA) is a type II transmembrane protein strongly overexpressed in most prostate cancer (PCa) cells, although also normally expressed in benign prostate tissue at lower levels (171, 172). In addition to prostate cancer, several studies have demonstrated increased PSMA expression in other malignancies, such as lung cancer, colorectal cancer, renal cell carcinoma, and BC (173, 174). Several case reports have shown avid PSMA uptake by both incidentally detected male breast cancer primary and metastases (175–184), but also false-positive uptake associated with gynecomastia. In 2017, Sathekge et al. (185) reported on imaging findings using [^68^Ga]PSMA-HBED-CC ([^68^Ga]Ga-PSMA-11) PET/CT in a series of 19 breast carcinoma female patients (9 treatment-naive patients, 5 patients with loco-regional recurrence and 5 patients before chemotherapy). Out of 81 tumor lesions identified with routinely performed staging examinations (including [^18^F]F-FDG PET/CT in 6/19 patients), 84% were identified on [^68^Ga]Ga-PSMA-11 PET/CT. No significant difference was found between [^68^Ga]Ga-PSMA-11 and [^18^F]F-FDG detection of lesions. No significant difference in PSMA uptake was found between progesterone receptor-positive and progesterone receptor-negative lesions. However, authors documented the absence of PSMA expression in normal vascular endothelium, as well as its limited expression on the luminal side of the intestinal epithelium. These results, although preliminary, appear promising for a possible future use of PSMA not only in a diagnostic but also in a theragnostic setting (radioligand therapy) in BC patients. In 2018, Tolkach et al. (182) reported the results of a compassionate treatment with [^177^Lu]Lu-PSMA performed in a 38-year-old woman with TNBC due to rapid progress after a wide range of systemic therapies. The [^177^Lu]Lu-PSMA treatment was approved after PSMA receptor status was demonstrated in [^68^Ga]Ga-PSMA-11 PET/CT. The patient obtained 7.5 GBq [^177^Lu]Lu-PSMA twice with a 4-week interval. The patient unfortunately progressed under therapy, but the low toxicity suggests further studies to elucidate the efficacy of PSMA-directed therapy, especially in cases of TNBC.

## Molecular Imaging of Immunotherapy (Immunopet)

Among the new therapeutic strategies for metastatic BC, immunotherapy has been gaining tremendous importance in recent years. Although more established in the treatment of other cancers, such as lung cancer (186) and melanoma, immunotherapy with immune-checkpoint inhibitors (ICIs) has recently acquired a predominant role in TNBC, which tends to be characterized by increased expression of programmed cell death protein 1 (PD-1), programmed cell death ligand 1 (PD-L1), a higher prevalence of tumor-infiltrating lymphocytes (TILs) and a higher mutational burden (187).

The Food and Drugs Administration (FDA) has approved combined chemotherapy and immunotherapy treatment with both anti PD-L1 (such as atezolizumab) and anti PD-1 (such as pembrolizumab) monoclonal antibodies for PD-L1+ TNBC (188). New indications for the use of these combination therapies are also expanding to other patient populations, such as advanced hormone receptor-positive (HR+) BC or HER-2 positive patients who are refractory to standard therapy (187).

Although the response to immunotherapy is very heterogeneous, patients who have achieved a response have a prolonged overall survival. Therefore, the main challenge is to develop and identify new biomarkers predictive of benefit from and resistance to immunotherapy. [^18^F]F-FDG is not able to assess the finer mechanisms underlying tumoral IC expression and resistance to immunotherapy. For this reason, new molecular imaging probes have been developed to improve our knowledge on the tumor microenvironment (TME), immune system and ICIs (189). New PET radiotracers targeting IC proteins may allow to systematically map PD-L1 tumor expression and/or PD-1 and cytotoxic T-lymphocyte associated protein 4 (CTLA-4) cell expression, in both spatial (whole-body) and temporal dimension, potentially providing added value for therapy and patient selection (186, 189).

In 2019, the first-in-human study by Bensch at al. (190) evaluated [^89^Zr]Zr-atezolizumab PET/CT performance in 22 patients with metastatic bladder cancer, non-small cell lung cancer (NSCLC), or TNBC. [^89^Zr]Zr-atezolizumab uptake was found to be physiologically high in bone marrow, intestine, kidneys, and liver, but low in brain, subcutaneous tissue, muscle, compact bone, and lung, while the uptake in lymph nodes and spleen depended on the activation state of the immune system. They found that pre-treatment radiotracer uptake better correlated with PFS and OS, compared to conventional IHC staining of PD-L1, highlighting the limitations of a single biopsy evaluation. Tumoral [^89^Zr]Zr-atezolizumab uptake was generally high (mean SUVmax of 10.4), but often with high intra-tumoral, inter-tumoral and inter-patient heterogeneity. Despite several new probes studied in the preclinical field, to date no other probes have been evaluated in humans with BC.

PET radiotracers with a labeled monoclonal antibody targeting the inducible T-cell costimulator (ICOS) could also gain an importance in this field, distinguishing tumor progression from pseudo-progression and identifying potential non-responding patients (patients which ICOS PET showing low or no uptake of activated T cells) and/or lesions. However, these radiotracers have yet not been studied in humans with BC (189).

Although immuno-PET radiotracers are still not entered clinical practice, in the future they may help clinicians to select patients who are good candidates for immunotherapy, identifying response earlier and potentially distinguish tumor progression from pseudo-progression.

## Fibroblast Activation Protein-α Targeted Radiotracers

As previously mentioned, the TME is a complex system of transformed tumor cells and other cellular and molecular components that regulate tumor development (189). In this context, cancer-associated fibroblasts (CAF) play an essential role in tumor growth, TME function, metastatic spread, and therapy resistance through potent immunosuppressive activity, conferring resistance to immune-based therapies (191).

In the last decade, researchers have attempted to better study CAF cells and their role in cancer evolution, which is correlated with T-cell immunosuppression and lead to poor clinical outcome (192), also by developing specific probes, such as new radiotracers targeting CAF-dependent pathways.

Fibroblast activation protein-α (FAPα), a transmembrane serine protease and marker of CAF activation, is overexpressed on CAF cell membrane and stroma in ~90% of epithelial neoplasms, whereas it is vastly absent in normal stromal cells (193). In 2018, researchers at the University of Heidelberg have introduced PET imaging of FAP expression in cancer, producing a [^68^Ga]-labeled FAP inhibitor (FAPi), derived from quinoline peptidomimetics, that binds with high affinity to FAP expressed on CAFs (194, 195). This new radiotracer seems extremely promising due to the excellent visualization of various kinds of tumors, owing to the high tumor-to-background ratio (TBR) (196, 197).

In 2021, two different studies (198, 199) compared [^68^Ga]Ga-FAPi PET/CT and [^18^F]F-FDG PET/CT in patients with BC. Elboga et al. (198) aimed to detect additional lesions in BC patients that may affect further chemotherapy options in 48 patients. The study demonstrated more lesions in all categorized regions in [^68^Ga]Ga-FAPi PET/CT with higher uptake compared to [^18^F]F-FDG PET/CT. In treatment response assessment of the post-chemotherapy group, [^68^Ga]Ga-FAPi PET/CT shifted 12 cases (12/24) from stable disease (SD) to progressive disease (PD) compared to [^18^F]F-FDG PET/CT, because of the assessment of lesions that were occult on [^18^F]F-FDG PET/CT. Kömek et al. (199) evaluated the assessment of primary tumor and metastases in 20 patients with histopathologically confirmed primary and recurrent BC. [^68^Ga]Ga-FAPi PET/CT was superior to [^18^F]F-FDG PET/CT in detecting breast lesions, as well as liver, bone, lymph node and brain metastases in terms of patient-based and lesion-based assessment, with higher uptake values compared to [^18^F]F-FDG PET/CT. The sensitivity and specificity of [^68^Ga]Ga-FAPi in detecting primary breast lesions were 100 and 95.6%, respectively, while [^18^F]F-FDG yielded 78.2 and 100%, respectively.

FAPi may also serve as theragnostic probes: the high TBR can be exploited by labeling FAPi with alpha- or beta-emitting isotopes to generate a potentially new intriguing radioligand therapy for BC, among other cancers (200–203).

## Gastrin-Releasing Peptide Receptor Targeted Radiotracers

Gastrin releasing peptide receptor (GRPR) belongs to subtype II of the bombesin receptor (BBN) family. It is a seven-transmembrane G protein-coupled receptor conjugated with BBN (204). The main physiological role of gastrin-releasing peptide (GRP) is the release of gastrin, contributing to the regulation of enteric function, but it may also hold a function in carcinogenesis and cell proliferation (205). Different studies have demonstrated GRPR overexpression in several cancer types, such as lung, gastric, pancreatic, prostate, colorectal, ovarian and endometrial cancer, and in glioma (206–208). Overexpression of GRPR has also been observed in BC, both in invasive ductal carcinoma (65%) and invasive lobular carcinoma (68%) (209).

Different molecular probes for visualizing GRPR expression have been developed (210, 211) and were subsequently used in preclinical and clinical studies. Notably, both agonists for GRPR and antagonists for GRPR have been developed. Particularly the antagonists have demonstrated improved image contrast and uptake compare to agonists, without adverse gastrointestinal effects (212).

Stoykow et al. (213) used [^68^Ga]Ga-RM2 PET/CT (RM2 is a GRPR antagonist) in patients referred for staging BC: an upstaging was found in 7/15 patients (47%) due to the detection of suspicious lymph nodes, owing to a favorable TBR. All PET-positive primary tumors were ER+ and PR+ (13/13) in contrast to only 1/5 PET-negative tumors, demonstrating that [^68^Ga]Ga-RM2 uptake correlates with ER expression in primary tumors of untreated patients.

More recently, Michalski et al. (214) assessed tumor binding of RM2 before and after neoadjuvant chemotherapy (NAC) in 7 primary BC, demonstrating a significantly reduced [^68^Ga]Ga-RM2 uptake on post-NAC PET/CT in all primary tumors. Moreover, the residual [^68^Ga]Ga-RM2 uptake in ER-positive primary BC correlated well with residual vital tumor size after NAC.

In 2021, a new study from the same group (215) on eight female patients with initial ER+ BC, demonstrated how [^68^Ga]Ga-RM2 PET/CT could support treatment decisions in these patients, guide radiotherapy planning in oligometastatic patients or select patients for RM2 radioligand therapy.

## Other New Radiotracers for Molecular Imaging in Breast Cancer

Several other radiotracers have been studied for BC assessment.

Neuroendocrine differentiation is observed in up to 20% of BC (216). BCs with neuroendocrine differentiation (NE BCs) are characterized by an overexpression of somatostatin receptors (SSTRs), as well as synaptophysin and chromogranin positivity (217). NE BCs may benefit from a non-invasive, whole-body PET/CT evaluation using SSTR radiotracers (218, 219) and in selected cases could also benefit from a theragnostic approach with peptide receptor radionuclide therapy (PRRT) (220, 221).

The study of proteins responsible for drug resistance has attracted the interest of many researchers in recent years, especially the study of P-glycoprotein (Pgp) (also known as multidrug-resistance protein 1 and ABCB1), which is a membrane protein able to actively remove drugs from the cell, with important implications in drug resistance in oncology (222). Studies of PET radiotracers, labeled with [^11^C] or [^18^F] and directed against Pgp are still in a preclinical phase. Nevertheless, tracers have been developed for both Pgp substrates and Pgp inhibitors (223). To date, only one feasibility study (dosimetric evaluation and tracer biodistribution assessment) was performed in 2011 by Kurdziel et al. (224), who administered [^18^F]-fluoropaclitaxel to 3 healthy volunteers and 3 patients with untreated BC (neoadjuvant chemotherapy candidates, tumor size > 2 cm), demonstrating the possible use of this radiotracer as a surrogate for paclitaxel.

Another biomarker of great interest in the field of molecular imaging is the imaging of apoptosis, which could help to drive therapeutic decisions, especially in the field of radiotherapy (225). The most studied tracers in this area target:

(1) annexin V, which detects phosphatidylserine expression on the cell surface. However, annexin V radiotracers have not performed well, mainly due to high background owing to slow blood clearance (226, 227);

(2) 2-(5-fluoro-pentyl)-2-methyl-malonic acid (ML-10), a lower molecular weight probe, labeled with [^18^F] (228);

(3) caspases, cysteine-aspartate-specific proteases, activated by intrinsic and extrinsic apoptotic pathways. A common treatment strategy is the induction of apoptosis in tumors, *via* the activation of the caspase cascade. Particular efforts have been made in the study of caspase 3-related radiotracers (229), such as [^18^F]F-ICMT-11, which has already provided promising results in BC patients (230).

Another field of growing interest is acetate radiotracers. Under metabolic stress, acetate becomes the main metabolic substrate for cancer cells instead of glucose. Acetate provides lipids and fatty acids and is particularly used by BC cells, which internalize it. Afterwards, acetate therefore binds to Co-A by acetyl-CoA synthetase (ACSS, EC 6.2.1.1), forming acetyl-CoA. It is precisely ACSS and its inhibitors that have been used to create radiotracers for acetate metabolism (231–233).

## Discussion

Breast cancer research is focusing on developing new drugs, that work only in tumors with a certain biomarker or mutation (2, 8). This has paved the way for improved survival of BC patients, assuming better individualization of the correct therapy for each tumor in each patient (personalized therapy) and assuming a prompt shift in therapy as the molecular and behavioral characteristics of the tumor change (temporal heterogeneity). In this scenario, PET imaging of ER and PR expression receptors is an attractive solution to the caveat of pathologic assessment by IHC and has an essential role in identifying patients who are candidates for hormone therapy and determining response to treatment. In addition, acquired resistance is a hallmark of endocrine therapy, and PET could be used to optimize clinical decision-making when resistance is acquired (234). Similarly, PET imaging of HER2 can provide important non-invasive, whole-body information about the tumor expression of this receptor. Indeed, as mentioned above, the development of HER2-specific therapies (such as trastuzumab) has resulted in improved outcomes in patients with HER2-positive tumors considered to have a poor prognosis (12). Moreover, dual PET imaging of ER and PR receptors' ligands combined with metabolic imaging ([^18^F]F-FDG), or better imaging of aggressive tumors (such as HER2 imaging or PSMA imaging) could improve assessment of tumor aggressiveness, allowing easier identification of targeted therapy for each patient.

These new radiopharmaceuticals may also provide an additional patient assessment tool for de-escalation, which is currently gaining interest in breast cancer not only in the surgical setting but also in terms of discontinuation of systemic therapy and reduction/interruption of radiotherapy treatment (8). In the balance between an acceptable increase in the risk of relapse and a potential decrease in side effects, radiopharmaceuticals such as FAPi, PSMA, and ligands of the ER, PR, and HER2 receptors can support the choice of de-escalation, providing additional information on the assessment of response compared with [^18^F]F-FDG, both in case of false-negative [^18^F]F-FDG PET (related to FDG-avidity of tumor type) and false-positive [^18^F]F-FDG PET (related to post-therapy changes, especially after radiotherapy).

Finally, the development of these new radiopharmaceuticals has introduced the theragnostics concept into the BC field, increasing the possibility of more personalized treatments based on individual and tumor characteristics soon (235–238).

## Conclusion

In recent decades, the advancement of modern medicine through multidisciplinary and translational approaches has contributed to a better understanding of the mechanisms and molecules involved in the development of breast cancer and responsible for its genotypic and phenotypic heterogeneity.

In nuclear medicine, these discoveries have allowed the development of new diagnostic biomarkers capable of assessing *in-vivo* and non-invasively several key features of breast cancer that are relevant for diagnosis, staging and restaging. These new probes can support clinical decision-making and patient selection by evaluating specific therapeutic targets for an improved assessment of response to specific treatments.

Molecular imaging is becoming an indispensable tool to support collaboration among health professionals involved in the fight against BC in order to achieve a more personalized therapy, through an increasingly accurate characterization of lesions using probes that can refine the ability to predict prognosis and response to therapy.

It is expected that these probes will be usable in the future also for therapeutic purposes owing to an increasingly prudent use of therapeutic radionuclides and the development of radioligand therapy.

## Data Availability Statement

The original contributions presented in the study are included in the article/supplementary material, further inquiries can be directed to the corresponding author.

## Author Contributions

MiB, VL, and DD: substantial contributions to the conception or design of the work. MiB, VL, MR, and RL: literature search and article selection. MiB and VL: drafting of the manuscript. MR, RL, MaB, AmB, DN, SP, AnB, GA, NQ, RA, SM, CD'A, ET, MH, AP, and DD: critical revision of the manuscript for important intellectual content. MiB, VL, RA, SM, CD'A, ET, MH, AP, and DD: supervision. All authors have read and agreed to the published version of the manuscript. All authors contributed to the article and approved the submitted version.

## Funding

MH is a recipient of grants from GE Healthcare, grants for translational and clinical cardiac and oncological research from the Alfred and Annemarie von Sick Grant legacy, and grants from the Artificial Intelligence in oncological Imaging Network by the University of Zurich.

## Conflict of Interest

The authors declare that the research was conducted in the absence of any commercial or financial relationships that could be construed as a potential conflict of interest.

## Publisher's Note

All claims expressed in this article are solely those of the authors and do not necessarily represent those of their affiliated organizations, or those of the publisher, the editors and the reviewers. Any product that may be evaluated in this article, or claim that may be made by its manufacturer, is not guaranteed or endorsed by the publisher.

## Abbreviations

[^11^C]C-choline: N-[^11^C]methyl-choline
[^11^C]C-MET: L-methyl-[^11^C]-methionine
[^18^F]F-FACBC: anti-1-amino-3-[^18^F]-fluorocyclo-butane-1-carboxylic acid
[^18^F]F-FASu: [^18^F]-5-fluoro-aminosuberic acid
[^18^F]F-FAZA: [^18^F]-fluoroazomycin-arabinoside
[^18^F]F-FDG: 2-deoxy-2-[^18^F]fluoro-D-glucose
[^18^F]F-FENP: 21-fluoro-16α-ethyl-19-norprogeserone
[^18^F]F-FES: 16α-[^18^F]-fluoro-17β-estradiol
[^18^F]F-FETE: 1-(-2(-2(-2[^18^F]fluoroethoxy)ethoxy)ethyl)-1H-1,2,3-triazole-estradiol
[^18^F]F-FETNIM: [^18^F]-fluoroerythronitroimidazole
[^18^F]F-FFNP: 21-^18^F-fluoro-16α, 17α-[(R)-(1′-α-furylmethylidene)dioxy]-19-norpregn-4-ene-3, 20-dione
[^18^F]F-FGln: [^18^F]-(2S,4R)4-fluoroglutamine
[^18^F]F-FLT: [^18^F]-fluoro-3′-deoxy-3′-L-fluorothymidine
[^18^F]F-FMISO: [^18^F]fluoromisonidazole
[^18^F]F-FSPG: (4S)-4-(3-[^18^F]-fluoropropyl)-l-glutamate
[^18^F]F-HX4: [^18^F]-3-fluoro-2-(4-((2-nitro-1H-imidazol-1-yl)methyl)-1H-1,2,3-triazol-1-yl)propan-1-ol
[^18^F]F-ISO-1: N-(4-(6, 7-dimethoxy-3,4-dihydroisoquinoline-2(1H)-yl)butyl)-2-(2-[^18^F]-fluoroethoxy)-5-methylbenzamide
[^18^F]F-RP-170: [^18^F]-1-(2-1-(1H-methyl) ethoxy)-methyl-2-nitroimidazole
4FM-[^18^F]F-FES: 4-fluoro-11β-methoxy-16α-[^18^F]-fluoroestradiol
ABCB1: multidrug resistance protein 1
BBN: bombesin receptor
BC: breast cancer
BRCA1: breast cancer gene 1
BRCA2: breast cancer gene 2
CAF: cancer associated fibroblasts
CAP: college of american pathologists
CT: computer tomography
CTLA-4: cytotoxic T-lymphocyte–associated protein 4
Cu-ATSM: non-nitroimidazole compound copper(II)diacetyl-bisN(4)-methylthiosemicarbazone
DCIS: ductal carcinoma *in situ*
EGFR: epidermal growth factor receptor
ENT1: equilibrative nucleoside transporter 1
ER: estrogen receptor
FAPα: fibroblast activation protein-α
FDA: food and drug administration
FMAU: 1-(29-deoxy-29-fluoro-b-D-arabinofuranosyl) thymine
GLUTs: glucose transporters
GRP: gastrin releasing peptide
GRPR: gastrin releasing peptide receptor
HER2: human epidermal growth factor receptor 2
HIF-1: hypoxia inducible factor 1
IBC: invasive breast cancer
IBC-NST: invasive breast cancer of no special type
ICIs: immune checkpoint inhibitors
ICOS: inducible T-cell costimulator
IDC: invasive ductal carcinoma
IHC: immunohistochemistry
ILBC: invasive lobular breast carcinoma
ISH: *in situ* hybridization
LHRH: luteinizing hormone-releasing hormone
ML-10: 2-(5-fluoro-pentyl)-2- methyl-malonic acid
MRI: magnetic resonance imaging
NAC: neoadjuvant chemotherapy
NE BCs: neuroendocrine differentiation breast cancers
NSCLC: non-small cell lung cancer
OS: overall survival
PCa: prostate cancer
PD-1: programmed cell death protein 1
PD-L1: programmed cell death ligand 1
PET: positron emission tomography
PFS: progression free survival
Pgp: P-glycoprotein
PR: progesterone receptor
PRRT: peptide receptor radionuclide therapy
PSMA: prostate specific membrane antigen
RGD: arginine-glycine-aspartic acid
SBR: scarff-bloom-richardson
SSTR: somatostatin receptors
SUV: standardized uptake value
TBR: tumor-to-background ratio
TILs: tumor-infiltrating lymphocytes
TK-1: thymidine kinase-1
TK-2: thymidine kinase-2
TME: tumor microenvironment
TNBC: triple negative breast cancer
VEGF: vascular endothelial growth factor
WHO: world health organization.
